# Umbilical Drainage in a Young Adult: Presentation and Management of an Infected Urachal Remnant

**DOI:** 10.7759/cureus.107829

**Published:** 2026-04-27

**Authors:** Joshua Chait, Arsha Sharma, Rachel E Bridwell, Zachary M Keene, Robert J Conrad

**Affiliations:** 1 General Surgery, Carolinas Medical Center, Charlotte, USA; 2 General Surgery, Wake Forest School of Medicine, Charlotte, USA; 3 Emergency Medicine, Carolinas Medical Center, Charlotte, USA; 4 Emergency Medicine, Army Special Operations Aviation Command, Fort Bragg, USA

**Keywords:** incision and drainage of abscess, infected urachal cyst, infected urachal remnant, umbilical abscess, urachal remnant

## Abstract

Urachal remnants are rare congenital anomalies that typically regress in early childhood, infrequently encountered in adults. When symptomatic, infection is the most common presentation and may mimic more common causes of abdominal pathology, leading to delayed diagnosis. We report the case of a 20-year-old male who presented with abdominal pain and purulent umbilical discharge that failed outpatient antibiotic therapy. Computed tomography (CT) demonstrated subcutaneous collections with calcified material concerning for an infected urachal remnant, confirmed on CT cystogram, requiring incision and drainage with drain placement, followed by planned definitive surgical excision. Because of the infrequent nature of adult urachal remnants, they may be underrecognized at initial presentation. CT is essential for diagnosis and operative planning, as parenteral antibiotics and drainage are the mainstay of treatment for acute infection, with delayed definitive excision, and are key to optimal outcomes and recurrence prevention.

## Introduction

Urachal remnants are a rare congenital anomaly, occurring in 1.6% of children under 15 years of age and in 0.063% of adults [1]. The urachus is a remnant of the obliterated allantois, the drainage duct of the fetal urinary bladder [2-4]. During normal development, descent of the bladder into the fetal pelvis during the third trimester results in obliteration of the urachus, forming the median umbilical ligament [2,5-6]. Failure of complete closure after six months of age predisposes to urachal pathology, most commonly identified in pediatric patients. In a retrospective study of 394 children undergoing laparoscopic inguinal hernia repair, urachal remnants were identified in 35.5%, with the majority detected in infancy [7]. Adult prevalence is lower because of spontaneous resolution in infancy and remains less well characterized. Failure of the tract to obliterate after birth can lead to a urachal remnant, urachal cyst, patent urachus, urachal sinus, and vesicourachal diverticulum [8]. Urachal abnormalities are most commonly asymptomatic; however, if infection occurs, it can lead to pain, dysuria, purulent or watery drainage, and fever [9-10]. We present the case of a 20-year-old male patient presenting with abdominal pain and umbilical drainage, found to have an infected urachal remnant requiring staged surgical management.

## Case presentation

**Figure 1:**
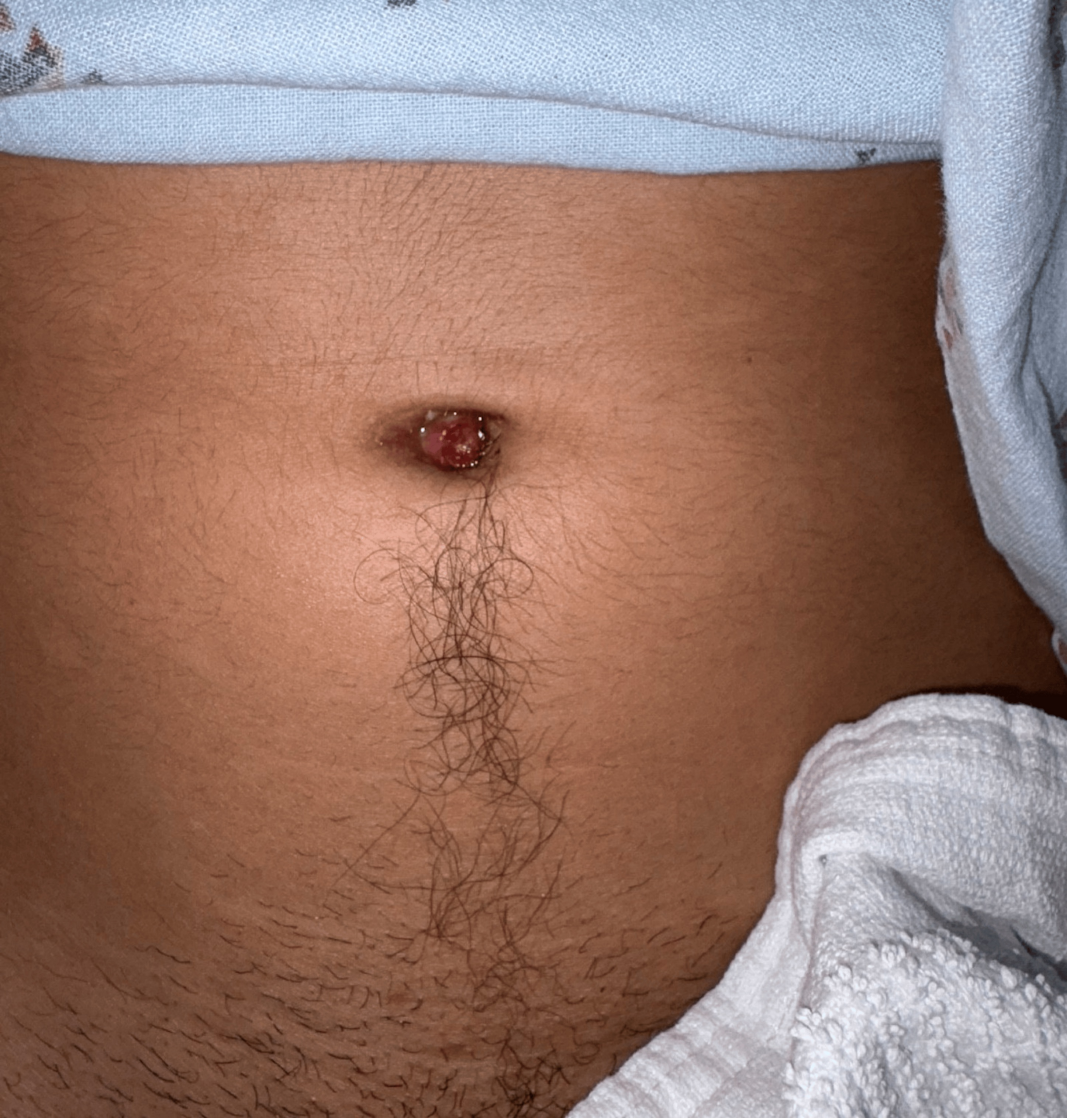
Patient’s umbilicus at initial presentation with umbilical granuloma.

**Figure 2:**
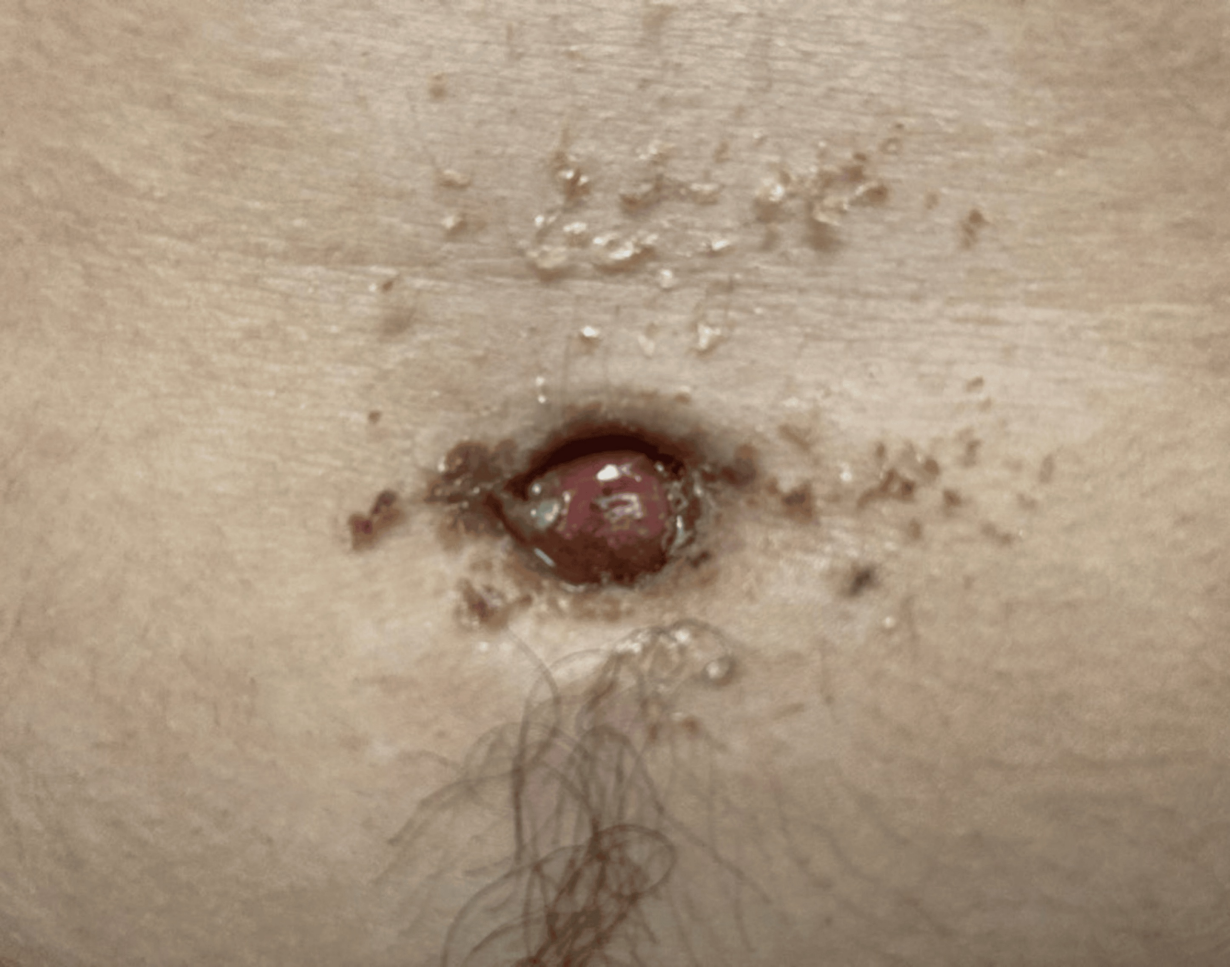
Patient’s umbilicus at initial presentation with inflammation and purulent drainage of the umbilicus.

A 20-year-old male with no significant past medical and surgical history initially presented to the emergency department (ED) with a 4-day history of abdominal pain and purulent umbilical discharge. The patient was initially discharged home on a 7-day course of 500 mg of cephalexin with the presumptive diagnosis of a subcutaneous abscess at the umbilicus. He presented to the ED four days later with continued pain and drainage around the umbilicus despite oral antibiotics. The patient endorsed a one-week history of intermittent dysuria, which had previously resolved, but denied fevers, chills, nausea, and emesis. He reported continued purulent drainage, redness, and pain around the umbilicus. On presentation, the patient’s temperature was 97.7ºF, heart rate 67 beats/min, and blood pressure 112/46 mmHg. On examination, the patient had an umbilical granuloma with expressible purulent drainage and tenderness (Figures 1, 2).

**Figure 3:**
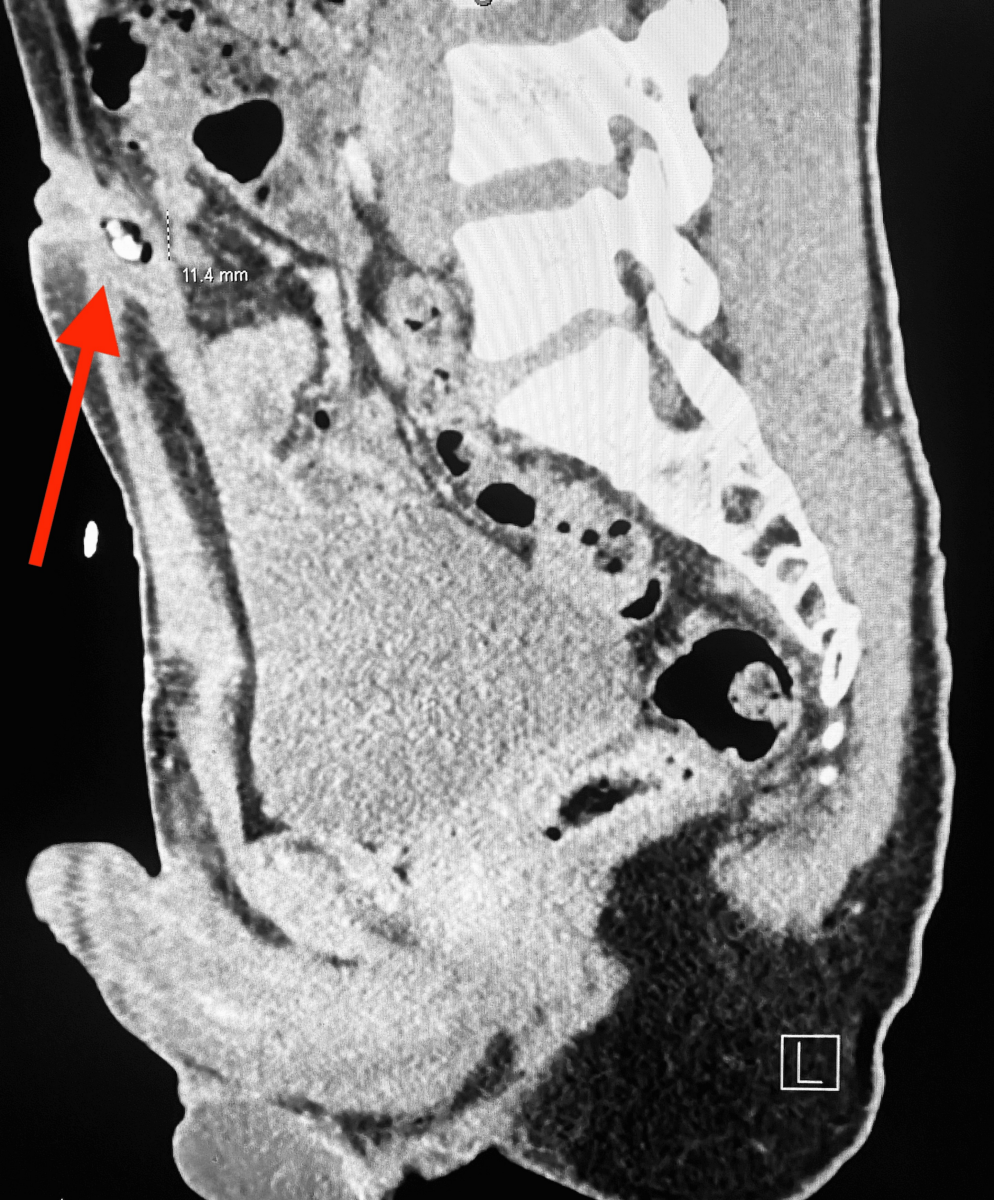
CT of the abdomen and pelvis Computed tomography (CT) images revealed skin thickening in the umbilicus with a subcutaneous central area of mixed attenuation approximately 1.7 cm deep to the skin surface measuring 1.3 x 1.1 x 1.1 cm and a smaller cutaneous collection with similar attenuation measures 0.6 x 0.5 x 0.7 cm, both containing calcified material.

Complete blood count (CBC), basic metabolic panel (BMP), and urinalysis were unremarkable, though he had a mildly elevated C-reactive protein at 0.6 mg/dL (upper limit of normal <0.5). The initial leading diagnosis on the differential was a subcutaneous umbilical abscess. Given the umbilical granuloma and failure of oral antibiotics, the differential diagnosis was more concerning of omphalitis and infected urachal abnormality. Computed Tomography (CT) was ordered by the emergency department to rule out other intra-abdominal pathology, such as an incarcerated hernia. CT of the abdomen and pelvis revealed skin thickening at the umbilicus with small subcutaneous collections containing calcified material consistent with an infected urachal remnant (Figure 3).

**Figure 4:**
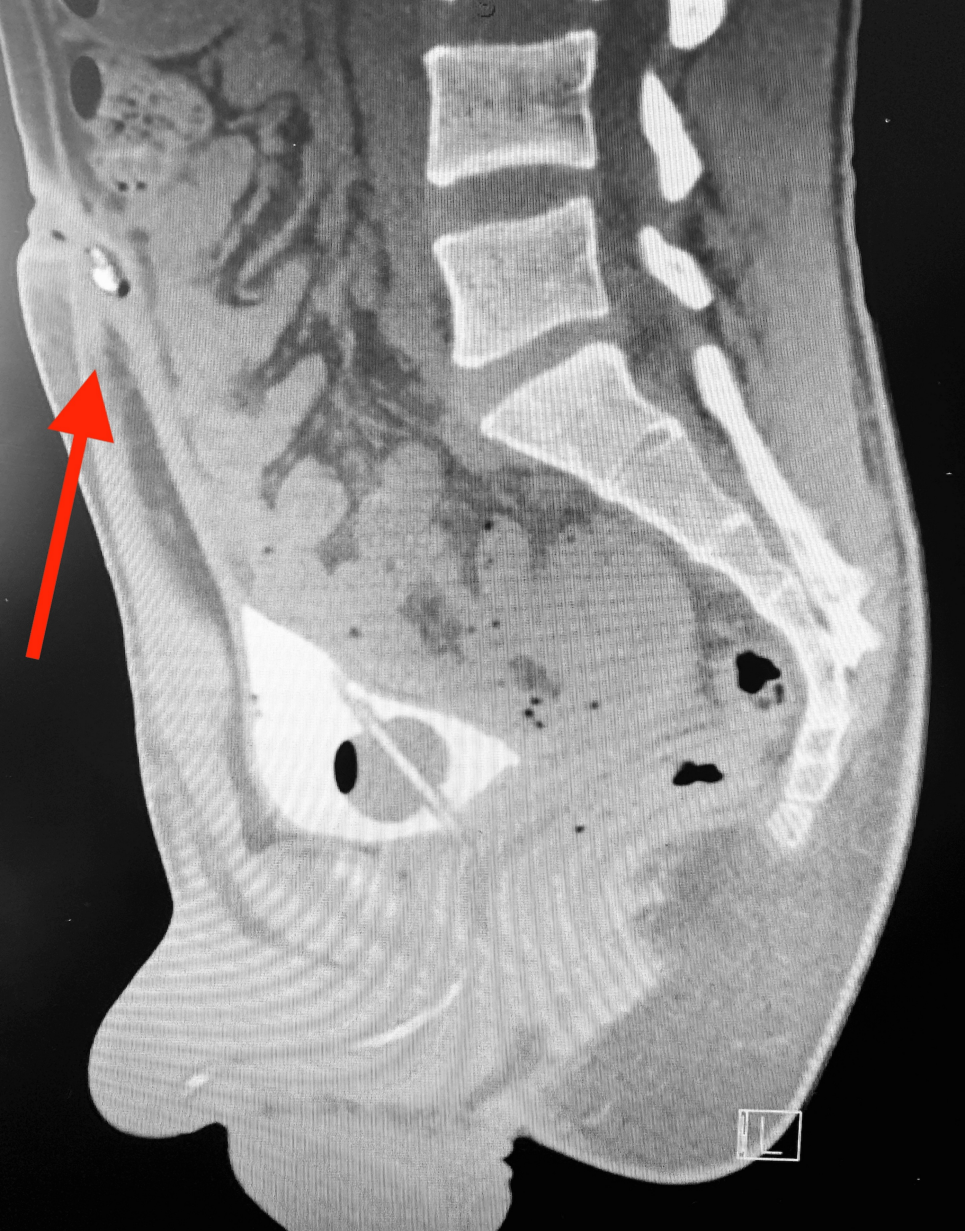
CT cystogram of the patient CT cystogram image showing a fibrous urachal remnant without evidence of a patent urachus or urachal cyst.

The patient was admitted to our general surgery service and started on 2 g intravenous ceftriaxone daily. Prior to operative management, the patient underwent a CT cystogram, which showed a fibrous urachal remnant without evidence of a patent urachus (Figure 4). The patient underwent an incision and drainage given active and persistent infection of the urachal remnant with penrose drain placement. Intraoperative exploration revealed a punctate opening within the inferior umbilicus into the urachal remnant, with calcified debris present in the sinus tract, confirmed on pathology. The patient had an otherwise uncomplicated postoperative course and was discharged on postoperative day one to follow up with urology for a robotic urachal remnant excision.

## Discussion

The four types of urachal remnants can be differentiated as patent urachus, umbilical-urachal sinus, urachal cyst, and vesicourachal diverticulum [11]. A patent urachus maintains bladder-umbilical continuity with possible urinary discharge. Umbilical-urachal sinuses and vesicourachal diverticula are non-communicating blind tracts. Conversely, urachal cysts are midline, sealed, fluid-filled lesions commonly found near the bladder dome [12]. The pathology described in our case presentation was most consistent with an umbilical-urachal sinus presenting with omphalitis, given the CT findings showing a urachal remnant and intraoperative findings of calcified debris within a sinus tract at the umbilicus.

Urachal remnant complications commonly present with lower abdominal pain, palpable mass, dysuria, urinary tract infection, or umbilical drainage. In a review of over 800,000 patients, 24 pediatric and adult patients with urachal remnants were identified; 13 (54.2%) presented with umbilical drainage and 17 (70.8%) required operative repair [13]. Adults typically present with suprapubic pain, dysuria, hematuria, or a palpable midline mass, whereas umbilical drainage is uncommon and usually indicates infection or a persistent urachal tract [13]. Infection of a urachal cyst may present acutely with fever and localized tenderness [14]. The clinical course in adults is variable; infection produces an acute/subacute presentation over days to weeks, whereas noninfected remnants may result in chronic, nonspecific symptoms persisting for months [14]. Laboratory markers such as CBC, C-reactive protein, erythrocyte sedimentation rate (ESR), and urinalysis may support suspicion for infection but are nonspecific [13,14]. Infected urachal cysts or abscesses are commonly initially mistaken for incarcerated umbilical hernias and acute appendicitis, with the correct diagnosis only established after advanced imaging such as CT or ultrasound [15,16].

Imaging is essential for the identification and operative planning of urachal remnant pathology. Ultrasound serves as a useful initial modality to confirm the presence of an umbilical fluid collection, while CT provides superior anatomic detail for complicated lesions and detecting solid components, calcifications, or local invasion [3,4]. Both urachal cysts and urachal carcinomas often appear similarly on imaging, showing increased echogenicity on ultrasound and thick-walled cystic or mixed-attenuation features on CT, making the distinction between them challenging [17]. MRI is reserved for lesion characterization or surgical planning when CT is indeterminate. Because imaging features of infection and malignancy often overlap, tissue diagnosis is frequently required when malignancy is a concern [6,16-17]. Once the urachal remnant anatomy is delineated on imaging, operative management varies depending on the presence of acute infection.

Surgical excision is considered the standard treatment for urachal remnants [6,18]. When patients present with acute infection of a urachal remnant, definitive excision should be delayed, and the acute infection should first be treated with antibiotics and aspiration or incision and drainage of purulent fluid if present. However, infected urachal remnants have a high risk of recurrence, up to 30% [14-18]. Urachal remnant excision had previously been performed open, but is now commonly performed minimally invasively using laparoscopic and robotic techniques [19]. Minimally invasive techniques are preferred due to decreased postoperative pain and better cosmetic outcomes, and there are several case reports describing robotic excision techniques [19]. The case findings align with the current literature on infected urachal remnants regarding presentation, diagnosis, and subsequent treatment.

## Conclusions

Urachal remnants are rare in adults but may present with acute infection with associated abdominal pain and umbilical drainage. Due to their nonspecific presentation, diagnosis may be delayed or mistaken for more common abdominal wall or intra-abdominal pathology. Ultrasound and cross-sectional imaging play a critical role in diagnosis, differentiation from malignancy, and surgical planning. Management of infected urachal remnants should follow a staged approach, with initial control of infection through antibiotics and surgical drainage, followed by definitive surgical excision to minimize recurrence. This unique case highlights a critical though unusual entity for clinicians to identify for proper diagnosis and operative management.
